# Mental Health and School Functioning for Girls in the Child Welfare System: the Mediating Role of Future Orientation and School Engagement

**DOI:** 10.1007/s12310-017-9207-6

**Published:** 2017-01-11

**Authors:** Jennifer M. Threlfall, Wendy Auslander, Donald Gerke, Hollee McGinnis, Sarah Myers Tlapek

**Affiliations:** 1 Department of Social Policy and Social Work, University of York, Heslington, York, YO10 5DD UK; 2 George Warren Brown School of Social Work, Washington University in St. Louis, Campus Box 1196, St. Louis, MO 63130 USA; 3 School of Social Work, University of Missouri, Columbia, MO 65211 USA

**Keywords:** School engagement, Future orientation, Depression, PTSD, Child welfare, Adolescence

## Abstract

This study investigated the association between mental health problems and academic and behavioral school functioning for adolescent girls in the child welfare system and determined whether school engagement and future orientation meditated the relationship. Participants were 231 girls aged between 12 and 19 who had been involved with the child welfare system. Results indicated that 39% of girls reported depressive symptoms in the clinical range and 54% reported posttraumatic symptoms in the clinical range. The most common school functioning problems reported were failing a class (41%) and physical fights with other students (35%). Participants reported a mean number of 1.7 school functioning problems. Higher levels of depression and PTSD were significantly associated with more school functioning problems. School engagement fully mediated the relationship between depression and school functioning and between PTSD and school functioning, both models controlling for age, race, and placement stability. Future orientation was not significantly associated with school functioning problems at the bivariate level. Findings suggest that school engagement is a potentially modifiable target for interventions aiming to ameliorate the negative influence of mental health problems on school functioning for adolescent girls with histories of abuse or neglect.

## Introduction

A surprisingly high number of students in schools in the USA are victims of abuse or neglect. Recent prevalence rates indicated that one in eight children was identified as a victim of abuse or neglect by the time they were 18, and girls were more likely to be victims than boys (Wildeman et al., 2014). Youth who are involved with the child welfare system due to experiences of abuse or neglect perform more poorly over a range of school-related domains, including behavior and academic performance (McMillen, Auslander, Elze, White, Thompson, 2003; Perfect, Turley, Carlson, Yohanna, & Saint Gilles, 2016). The poor school functioning of child welfare-involved youth has been explained in part by the high rates of mental health problems in the population, resulting from their histories of abuse and neglect (Romano, Babchishin, Marquis, & Fréchette, 2015). However, the mechanisms by which mental health problems may affect school outcomes are not well understood, either in the general population or among child welfare-involved youth. The current study explored two potential pathways—future orientation and school engagement—through which mental health may impact school functioning for adolescent girls involved in the child welfare system.

Children and their families usually become involved in the child welfare system following an investigation of suspected abuse or neglect. In 2014, investigations in the USA by child protective services (CPS) identified 702,000 victims [U.S. Department of Health and Human Services (DHHS), 2016a]. The majority of youth who received child welfare services following a CPS investigation remained in their homes; around a fourth received an out-of-home placement (DHHS, 2016a). In 2015, 45% of youth who had been removed from their homes were placed in non-relative foster families and 35% were fostered by their relatives. Other youth lived in a group home or institution (14%) or were in a pre-adoptive home (4%) (DHHS, 2016b). Both youth who remain in home and those in out-of-home placements report higher levels of mental health and school functioning problems than youth in the general population (Heneghan et al., 2013; Perfect et al., 2016).

Child welfare-involved adolescents are especially vulnerable to mental health problems such as depression and posttraumatic stress disorder (PTSD) that may result from lifetime experiences of abuse or neglect (Heneghan et al., 2013; Kolko et al., 2010; McMillen et al., 2005; Turner, Finkelhor, & Ormrod, 2006). Among youth involved in the child welfare system, girls are more likely to experience mental health problems than boys. For example, an analysis of the National Survey on Child and Adolescent Well-Being (NSCAW) found that around 37% of adolescent boys and 47% of adolescent girls who were investigated by CPS reported at least one mental health problem (Heneghan et al., 2013). Studies of child welfare-involved adolescents in out-of-home care have reported similarly high prevalence rates of mental health problems. A study of older adolescents leaving the foster care system in three Midwestern states found that 11% of all youth and 14% of girls were assessed as having experienced major depression over their lifetime, and 15% of all youth and 22% of girls met the lifetime criteria for PTSD (Keller, Salazar, & Courtney, 2010).

The high prevalence of mental health problems among child welfare-involved youth has been associated with negative outcomes, such as poor academic and behavioral school functioning (Romano et al., 2015). Youth who are involved in child welfare have consistently been found to have lower achievement and more school-related behavioral problems than the general population, as evidenced through grade point averages, performance on standardized tests, and grade retention (Perfect et al., 2016; Romano et al., 2015). Furthermore, they are more likely to engage in problematic behaviors such as absenteeism and defiance and aggression in school (Pears, Kim, Fisher, & Yoerger, 2013; Perfect et al., 2016). Despite this overwhelming evidence that child welfare-involved youth have high rates of both mental health problems and poor school functioning, few studies have examined how these factors may be related to each other among child welfare-involved youth. In the general population, however, there is mounting evidence of the relationship between mental health problems and poor school functioning.

Mental health problems can affect both academic and behavioral outcomes. Students with internalizing problems such as anxiety and depression have demonstrated lower levels of achievement and attainment (Duchesne, Vitaro, Larose, & Tremblay, 2008; Fergusson & Woodward, 2002). They are also more likely to engage in problem behaviors at school such as aggression, bullying, and truancy (Ek & Eriksson, 2013; Ferguson, San Miguel, & Hartley, 2009). Posttraumatic symptoms following exposure to violence have also been found to have a negative impact on academic performance as indicated by standardized test scores, grade point averages, and grade retention (Lipschitz, Rasmusson, Anyan, Cromwell, & Southwick, 2000; Mathews, Dempsey, & Overstreet, 2009). Similarly, posttraumatic symptoms have been associated with externalizing behaviors and violence and aggression in school, as well as more frequent suspensions (Gellman & DeLucia-Waack, 2006; Lipschitz et al., 2000; Saigh, Yasik, Oberfield, Halamandaris, & McHugh, 2002).

Despite the growing evidence of the negative impact of mental health problems on school functioning, little is known about linkages between the two, either in the general population or among child welfare-involved youth. To deepen our understanding of the pathways by which mental health problems may be linked to poor school functioning, theory related to developmental assets was examined. Developmental assets have been defined as resources that youth possess internally or have access to in their environments that make successful functioning more likely (Lerner, Lerner, von Eye, Bowers, & Lewin-Bizan, 2011). Within the school context, adolescents who are in possession of the appropriate developmental assets may be expected to engage in constructive learning and social behaviors and to experience academic success. A broad range of assets have been identified that are associated with positive school functioning, including interpersonal strengths (e.g., supportive teachers, family, and community), and individual strengths (e.g., beliefs and values about schooling and the future) (Li, Lerner, & Lerner, 2010; Scales, Benson, Roehlkepartain, Sesma, & Dulmen, 2006).

Previous research has suggested the importance of developmental assets in promoting positive outcomes among younger children involved in child welfare. For example, internal developmental assets such as a commitment to learning and sense of purpose have been associated with fewer conduct problems and higher educational performance in children living in out-of-home care (Bell, Romano, & Flynn, 2015; Filbert & Flynn, 2010). Less is known about the role of developmental assets for adolescents involved in child welfare. Moreover, previous studies have not examined developmental assets as mediators of the relationship between mental health problems and school functioning.

Thus, the focus of the current study was to examine the role of two developmental assets: school engagement and future orientation. Adolescents in the child welfare system may have difficulty functioning in school because the formation of these developmental assets has been interrupted. Specifically, the mental health problems often experienced by children with histories of abuse or neglect may prevent the formation of positive attitudes to school and the future that in other circumstances would lead to school success. In other words, developmental assets, such as school engagement and future orientation, are hypothesized to serve as pathways or mediators in the relationship between mental health problems and school functioning for adolescent girls in child welfare.

School engagement is a multidimensional concept that includes behavioral, cognitive, and emotional dimensions. The focus of the current study is on emotional engagement which has been defined as the extent to which students value their schooling, feel that they belong to their school, and are supported by their peers and teachers (Jimerson, Campos, & Greif, 2014). Research has demonstrated that emotional engagement was significantly associated with academic achievement in adolescents (Goodenow, 1993a; Wang & Holcombe, 2010) and fewer problem behaviors such as fighting and absenteeism (Catalano, Oesterle, Fleming, & Hawkins, 2004; Sánchez, Colón, & Esparza, 2005).

The negative impact of certain mental health on students’ emotional engagement has been demonstrated in studies of adolescents in the general population (Wang & Peck, 2012). Specifically, students who experience depression are less likely to have a strong sense of belonging to their school (Johnson, Crosnoe, & Thaden, 2006; Ma, 2003). The relationship between posttraumatic symptoms and school engagement is less clearly established. One study found that older adolescents with higher posttraumatic symptomatology had more negative attitudes toward their school and teachers (McGill et al., 2014).

Despite the overwhelming evidence that child welfare-involved youth face multiple problems with school functioning, only a few studies have explored school engagement in this population. In general, abused or neglected youth have been found to have lower levels of engagement with school when compared with youth of a similar socioeconomic background (Pears et al., 2013). Similarly to the general population, child welfare-involved youth who are more engaged in school have higher levels of achievement and have also been found to engage in fewer problem behaviors, such as fighting (Leonard, Stiles, & Gudiño, 2016; Pears et al., 2013). However, there is very little knowledge about the relationship between mental health and school engagement for child welfare-involved youth, or the role of school engagement as a mediator between mental health and school functioning.

Likewise, future orientation, defined as an individual’s expectations about and actions related to the future (Nurmi, 2005; Seginer, 2009), can be also considered as a developmental asset for adolescents. Youth who are more oriented toward the future believe that they have a certain amount of agency over their life trajectory and may make decisions that will maximize their chances of reaching goals they have set for themselves (Nurmi, 2005). Accordingly, higher levels of future orientation have been associated with greater academic achievement (Adelabu, 2007) and with a decrease in problem behaviors (Chen & Vazsonyi, 2013; Robbins & Bryan, 2004).

Mental health problems have been shown to have a negative effect on future orientation. Adolescents who show more depressive symptoms were less likely to have a positive orientation to the future (Kagan, MacLeod, & Pote, 2004). Furthermore, adolescents experiencing posttraumatic symptoms were more likely to have negative cognitions about the future (Allwood, Esposito-Smythers, Swenson, & Spirito, 2014). For example, one study found that urban youth who had been exposed to trauma and who had PTSD were more pessimistic about their futures than traumatized youth with no PTSD symptoms (Rialon, 2011). These established associations with school functioning and mental health problems support the possible role of future orientation as a pathway between the two in the general population.

A few studies have pointed to the role of future orientation as a protective factor for problem behaviors and mental health for children who have been abused or neglected (Edmond, Auslander, Elze, & Bowland, 2006; Herrenkohl, Tajima, Whitney, & Huang, 2005), but there has been little investigation of its relationship with school functioning. Moreover, some studies have cast doubt on the positive role of future expectations for child welfare-involved youth, finding no evidence of differences in risky behaviors for youth with differing levels of future orientation (James, Montgomery, Leslie, & Zhang, 2009; Traube, James, Zhang, & Landsverk, 2012). Because findings from previous research have been inconsistent, further research is warranted on the role of future orientation in promoting positive outcomes for child welfare-involved youth and as a potential mediator or pathway between mental health problems and school functioning.

To deepen our understanding of the developmental assets of girls involved in the child welfare system, the following research questions were addressed in the current study: (1) What are the associations between mental health problems (depression and PTSD) and school functioning for adolescent girls involved in the child welfare system? and (2) do developmental assets, such as school engagement and future orientation, mediate this relationship in this population?

## Method

### Participants

Participants were 231 adolescent girls who were involved in the child welfare system and who had been recruited to take part in a trauma-focused group intervention study. Baseline data collected for the intervention study were used for the current analysis. In order to be included in the study, girls needed to be between 12 and 19 years old and to have been formally investigated for child abuse or neglect through the state’s child protective services. Girls were excluded for the following criteria: if they were unable to read or write, had been hospitalized for mental health problems in the last 6 months, were unable to tolerate discussing abuse or neglect, or if they had behaviors that would preclude participation in a structured interview or group treatment. Participants were recruited through referrals from child protective services, other agencies providing services to adolescents in the child welfare system, and from caregivers.

The mean age of participants was 14.8 (SD = 1.6). Two-thirds of the girls were in high school (25% were seniors or juniors, and 41% were sophomores or freshmen). The remaining third were in middle school. Seventy-five percent of the girls were youth of color, of whom the majority (70%) identified as African-American and the others (5%) as Native American, Hispanic, Asian/Asian American, or “Other.” The remaining 25% of participants identified as non-Hispanic White. Participants were living with their biological parent(s) (40%), with another relative (14%), with a legal adoptive family (7%), with a foster family (28%), or in a group home or other congregate care facility (10%).

### Procedures

All study procedures were first approved by the Human Subjects Institutional Review Board of the two collaborating universities of study personnel. The Research Committee of the state office of child protective services also approved the research protocol. Additionally, a Certificate of Confidentiality was issued by the funding agency, the Centers for Disease Control and Prevention (CDC), in order to protect the privacy of the study participants.

Following the youth’s referral to the study team and confirmation of the adolescent’s expressed interest in the study, written consent was obtained from the legal custodian. In addition, to the fullest extent possible, written consent was also secured from all members of the youth’s family support team (e.g., guardian ad litem, deputy juvenile officer, child’s current therapist). All adolescents over the age of 18 provided written consent to participate in the study, and younger adolescents provided their written assent. Participants were given a $20 gift card to compensate them for their time.

Face-to-face interviews were conducted by masters or doctoral-level social work students. All interviewers participated in 8 h of training that focused on basic interviewing skills, confidentiality, procedures for reporting abuse and for responding in cases where participants endorsed items relating to suicide, as well as providing background knowledge about the population. Interviews lasted for approximately 1 h and were conducted in the participants’ homes or in community-based mental health agencies.

### Measures

#### Depression

Depressive symptoms over the previous 2 weeks were measured using the Child Depression Inventory (CDI; Kovacs, 2003). The CDI includes 27 items that are scored 0–2 and then summed, with higher total scores indicating more severe symptoms. Previous studies have demonstrated concurrent validity, distinguishing between normative and clinical groups (Kovacs, 2003; Saylor, Finch, Spirito, & Bennett, 1984). Furthermore, good internal consistency and test–retest reliability have been demonstrated for a child welfare population (Kolko et al., 2010). The internal consistency reliability for the current study was *α* = .88.

#### PTSD Symptoms

The Child PTSD Symptom Scale (CPSS; Foa, Johnson, Feeny, & Treadwell, 2001) was used to assess posttraumatic stress symptoms. Participants rated the frequency of experiencing symptoms over the past month for 17 items (e.g., Trying not to think about, talk about, or have feelings about the trauma; Having trouble falling or staying asleep) on a four-point scale from *not at all* (0) to *five or more times a week* (3). Previous studies have demonstrated that the CPSS has convergent validity, correlating highly with other similar PTSD scales, as well as good internal consistency and test–retest reliability (Foa et al., 2001). For the current sample, the internal consistency alpha coefficient was *α* = 0 .90.

#### School Engagement

School engagement was measured using seven items from the Psychological Sense of School Membership (PSSM; Goodenow, 1993b). The items assess students’ emotional engagement as indicated by their feelings about their school and about their relationships with teachers and peers. The items (e.g., I feel proud of belonging to my school; People at my school notice when I am good at something) were scored on a four-point scale from *strongly disagree* (1) to *strongly agree* (4) and then summed to form a total score. Higher levels of engagement are indicated by higher total scores. A previous study of foster care youth found high correlations in the expected direction between the seven-item scale and measures of achievement (e.g., grades and number of classes failed), establishing convergent validity (White, 2005). Internal consistency reliability was *α* = .78.

#### Future Orientation

Future orientation was assessed using a scale that measured two domains of future orientation. The affective domain (i.e., hope for the future) was assessed using six items from the revised version of the Life Orientation Test (LOT-R; Scheier, Carver, & Bridges, 1994) and the cognitive domain (i.e., thinking about the future) using four items from Heimberg’s Future Time Perspective Inventory (FTPI; Heimberg, 1963). Items were scored on a four-point scale from *strongly disagree* to *strongly agree* and then summed to form a total scale with a possible range of 10–40. Higher scores indicated a more positive future orientation. A previous study found significant differences in the scores of foster care youth and upper middle-class college-bound youth, thereby establishing the scale’s concurrent validity (Cabrera, Auslander, & Polgar, 2009). The alpha coefficient for the 10-item scale used in the current analysis was *α* = .71.

#### School Functioning Problems

The number of school functioning problems adolescents were experiencing was measured using an index of six items indicating behavioral or academic difficulties in school. Participants were asked to indicate whether they had participated in each of the four behavioral problems in the past academic year (*Yes/No*): skipping school, physical fights with other students, verbal fights with teachers, and physical fights with teachers. Academic problems were assessed by two items: “failed a class in the previous year” (*Yes/No*) and grades they had mostly received in that year (0 = *Mostly A’s*, *Mostly B’s*, and *Mostly C’s*; 1 = *Mostly D’s*, and *Mostly F’s*). Each problem that the participant endorsed was scored as one, resulting in an index with a possible range of 0–6 with higher scores indicating more school functioning problems.

#### Potential Control Variables

Several control variables that could potentially affect school functioning were investigated. These included age, race (0 = *youth of color*, 1 = *White*), placement instability (a count of the number of different types of placement that the adolescent had lived in during her lifetime), and school instability (number of school districts attended since sixth grade).

### Data Analysis

The potential mediating roles of school engagement and future orientation were explored to understand the pathways between mental health problems and school problems, using the steps outlined by Baron and Kenny (1986). First, correlations between the predictor, mediator, outcome, and potential control variables were examined. Second, where significant correlations were found, mediation analyses were conducted using the Hayes (2013) PROCESS SAS macro. The significance of the indirect effect (the impact of the depression and PTSD on school problems) through two potential mediators (school engagement and future orientation) was determined using a bootstrapping methodology provided by the Hayes macro (Preacher & Hayes, 2004).

## Results

### Descriptive Analyses

Results of the descriptive analyses, as shown in Table 1, indicated that the mean depression score of the participants was 11.71 (SD = 8.25). Based on previous studies of clinically referred adolescents using a clinical cutoff score of 13 and above (Kovacs, 2003), 39% of participants in the present study had depressive symptoms in the clinical range. Results from the PTSD scale indicated that the mean PTSD symptom score was 16.72 (SD = 10.83), with 54% of participants who reported PTSD symptoms in the clinical range (≥15).

**Table 1:** Means (M) and standard deviations (SD) of variables

Variable	M	SD
Depression	11.71	8.25
PTSD	16.72	10.83
School engagement	20.60	3.85
Future orientation	28.06	4.14
School functioning problems	1.37	1.20
Home instability	3.78	2.11
School instability	3.70	3.16

*N* = 231

*PTSD* posttraumatic stress disorder

The frequencies of participants endorsing each school functioning problem are shown in Table 2. The most common school functioning problem reported was failing a class (40.69%) followed by engaging in physical fights with other students (35.06%). Approximately one quarter of participants had skipped school (26.41%) and a similar proportion (25.11%) reported verbal fights with their teachers. Fewer students reported receiving lower overall grades (9.09%) or having engaged in physical fights with teachers (.87%). As shown in Table 3, the majority of participants (72.73%) reported experiencing at least one school functioning problem (*M* = 1.37, SD = 1.20).

**Table 2:** Frequencies of participants endorsing each school functioning problem

School functioning problems	*n*	%
Failed a class	94	40.69
Physical fights with students	81	35.06
Skipped school	61	26.41
Verbal fights with teachers	58	25.11
Low grades	21	9.09
Physical fights with teachers	2	.87

*N* = 231

**Table 3:** School functioning problems index frequencies

No. of school functioning problems	*n*	%
0	63	27.27
1	77	33.33
2	50	21.65
3	25	10.82
4	15	6.49
5	1	.43

*N* = 231

### Bivariate and Mediation Analyses

Bivariate relationships between the predictor, mediator, outcome, and control variables are shown in Table 4. Significant relationships were found between both mental health variables (depression and PTSD) and school functioning problems, with girls who reported higher levels of depression and PTSD symptoms having more school problems. Likewise, higher levels of depression and PTSD were significantly associated with lower levels of school engagement and lower levels of future orientation. Last, higher levels of school engagement were significantly associated with school problems; however, no relationship was found between future orientation and school problems. Results of the bivariate analyses indicated significant relationships between the control variables (age, race, and placement instability) and school functioning problems. Therefore, these variables were included as covariates in the mediation models.

**Table 4:** Bivariate correlations between mental health problems, developmental assets (school engagement, future orientation), and school functioning

Variable	1	2	3	4	5	6	7	8
1. School functioning	–							
2. Depression	.16^*^	–						
3. PTSD	.13^*^	.75^***^	–					
4. School engagement	−.28^***^	−.40^***^	−.19^**^	–				
5. Future orientation	−.06	−.58^***^	−.38^**^	.38^***^	–			
6. Age	.17^**^	.01	−.06	−.03	.10	–		
7. Race^a^	−.14^*^	.11	.05	−.04	−.11	−.02	–	
8. Placement instability	.13^*^	.06	.05	−.04	.03	.27^***^	−.06	–
9. School instability	.09	−.04	−.04	.01	.05	.35^***^	−.13^*^	.30^***^

*N* = 231

*PTSD* posttraumatic stress disorder

^a^0 = Youths of color, 1 = White

* *p* < .05; ** *p* < .01; *** *p* < .001

**Fig. 1:**
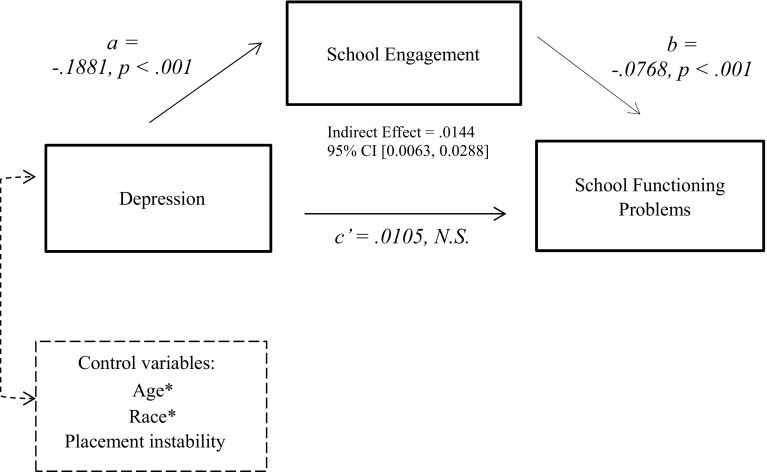
Mediating effect of school engagement on the relationship between depression and school functioning problems. *Significant covariate

**Fig. 2:**
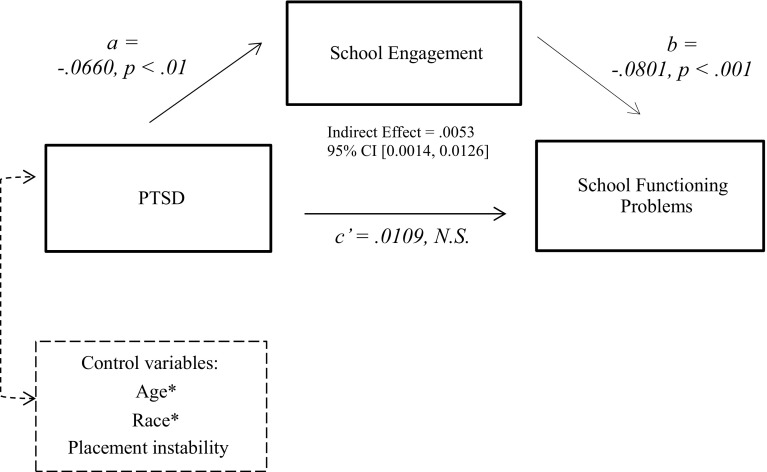
Mediating effect of school engagement on the relationship between PTSD and school functioning problems. *Significant covariate

Figure 1 displays the results of the mediation models testing the pathway between depression and school functioning problems through school engagement, controlling for age, race, and placement instability. Higher levels of depression were associated with more school functioning problems, and this relationship was fully mediated by school engagement. The bootstrapping analysis confirmed the mediating role of school engagement, indicating that the indirect path between depression and school problems was significant. Similarly, as shown in Fig. 2, the pathway between PTSD symptoms and school functioning problems became nonsignificant when school engagement was added to the model, controlling for age, race, and placement instability. More severe PTSD symptoms were associated with more school functioning problems, and the relationship was fully mediated by school engagement. The bootstrapping analysis confirmed a significant indirect path between PTSD symptoms and school functioning problems when school engagement is in the model. Because there was no significant bivariate relationship between future orientation (the other potential mediator) and school problems, the mediation model testing the indirect effects of mental health problems on school problems through future orientation was not tested.

## Discussion

The current study explored two possible pathways (i.e., through school engagement and future orientation) by which mental health problems influence school functioning for adolescent girls involved in child welfare. The results indicate that higher levels of depression and PTSD symptoms are significantly associated with greater school functioning problems and that school engagement fully mediated these relationships. The findings confirm our knowledge of the importance of depression and PTSD as predictors of school-related problems. Moreover, the findings identify a significant pathway (i.e., school engagement) by which mental health problems influence school functioning in child welfare-involved adolescent girls.

School engagement was conceptualized in the current study as a sense of belonging to school, including having positive relationships with teachers and peers. Findings suggest that that mental health problems, such as depression and PTSD, may negatively impact school engagement which, in turn, disrupts positive school functioning. This is consistent with previous studies that have shown that depression is predictive of later erosion in social support among adolescent girls, possibly because depressed individuals tend to engage in behaviors such as constantly seeking reassurance that may lead to peer rejection (Stice, Ragan, & Randall, 2004). Additionally, depressive symptoms may lead to a depletion of cognitive and emotional resources and a subsequent withdrawal from social relationships (Davis et al., 2016). Similarly, behaviors associated with PTSD symptoms, such as aggression, may undermine supportive relationships in school (Kendra, Bell, & Guimond, 2012). Another explanation is that mental health problems may also lead to cognitive distortions about the true nature of social relationships. For example, depressed youth have been found to have more negative conceptions of their social status than would be suggested by their teachers’ observations (Rudolph & Clark, 2001). Girls participating in the current study may therefore have underestimated the extent to which they are supported by their teachers and peers. The negative impact of diminished school engagement on school functioning in the current study is also consistent with other research. Students who do not feel supported by their peers and teachers and who do not have a strong sense of belonging to the school are unlikely to be motivated to engage in behaviors that are valued by that institution or that are conducive to their own academic success (Appleton, Christenson, & Furlong, 2008).

In contrast to some previous research, future orientation was not significantly related to school functioning at the bivariate level in the current study. Although many adolescents involved in child welfare have been found to have a strong orientation toward the future (Polgar & Auslander, 2009), current findings indicate that positive cognitions about the future and plans for the future do not always translate into behaviors that make educational success more likely. One explanation for this finding might be that child welfare-involved youth with mental health problems such as depression and PTSD lack other protective factors that have proved important for educational success in the general population. For example, youth who have histories of unstable home placements may not receive the consistent support of a caring adult that would enable them to engage in pro-educational behaviors that would lead to school success.

In interpreting the results of the study, some limitations should be considered. First, the data used in the analysis are cross-sectional. It is therefore not possible to draw conclusions about the causality of the relationships found. Second, the findings may not be generalizable to all adolescent girls with histories of abuse and neglect. Participants were a convenience sample recruited to take part in a trauma-focused group intervention and may differ from girls who did not need treatment, who were considered unable to participate, or who were simply not interested in group treatment. Third, all the items included in the school functioning problems index relied on the adolescents’ self-report of their achievement and behavior. Collateral information such as academic and disciplinary records obtained from their schools may have provided a more objective or valid indicator of school functioning. Although self-report methodologies are commonly used to assess school functioning among child welfare-involved youth (Perfect et al., 2016), little is known about their validity in this population. Therefore, further research should incorporate objective indicators to test the accuracy of these data.

The current study’s findings have important implications for the practice of professionals working with adolescent girls involved in child welfare within school settings. In particular, evidence about the role of school engagement as a pathway linking mental health problems to school functioning indicates the importance of considering the wider school environment in interventions with this population. Interventions that increase students’ school engagement may provide a way of interrupting the pathway by which mental health problems increase the likelihood of poor school functioning. Such interventions may specifically target youth who have experienced a trauma, such as abuse or neglect, or may alternatively be offered to all students in a school.

Interventions that specifically target students with histories of trauma, such as abuse or neglect, may be adapted to promote students’ school engagement in addition to other mental health and school-related outcomes. For example, interventions may be designed to be delivered by non-clinical professionals such as teachers and counselors who are permanently located in the adolescents’ schools. Students may thereby be given the opportunity to form positive ongoing relationships with these professionals, giving them a sense of being cared for and supported by adults within the school. There is some evidence that empirically supported trauma-focused treatments may be effectively implemented in schools by non-clinical professionals. For example, one intervention, *Support for Students Exposed to Trauma* (SSET; Jaycox et al., 2009), an adaptation of the Cognitive Behavioral Intervention for Trauma in Schools (CBITS; Jaycox, 2003), has been effectively delivered by teachers. Additionally, this intervention has also been implemented with girls involved in the child welfare system (Auslander et al., 2016).

It is unlikely that all adolescents who have been exposed to abuse or neglect will be identified for trauma-specific interventions. School-wide interventions that increase school engagement may therefore be a valuable means of supporting positive school functioning for these unidentified adolescents as well as for other students with undiagnosed or unaddressed mental health needs. An example is provided by universal socioemotional learning (SEL) curricula, which target students’ attachment, engagement, and commitment to their schools as a key means of promoting positive school functioning (Zins, Bloodworth, Weissberg, & Walberg, 2004). To achieve these goals, SEL programs focus on building supportive learning environments and strengthening relationships between students, their families, teachers, and other professionals. Increased opportunities for students to contribute to their class, school, and community may also increase their sense of belonging (Durlak, Weissberg, Dymnicki, Taylor, & Schellinger, 2011). By building school engagement, SEL programs and other similar interventions may therefore serve a preventative role in interrupting the pathway between mental health problems and poor school functioning for all adolescents, including those with child welfare involvement.

The findings of the current study contribute toward our theoretical understanding of how common sequelae of abuse and neglect can impact academic and behavioral outcomes. The major finding of the study that school engagement fully mediates the relationship between both depression and PTSD and school functioning for child welfare-involved girls highlights the need for further research about this important developmental asset. There is a critical need to deepen our understanding about how youth involved in child welfare who have symptoms of depression and PTSD can develop a sense of belonging to their schools, and how the school environment might be best shaped to provide resources and supportive relationships for these adolescents to promote positive outcomes in school. The results suggest the need for an educational system that encourages school engagement and educational achievement among girls in the child welfare system.
